# Editorial: Stem Cells in Neurodegeneration: Disease Modeling and Therapeutics

**DOI:** 10.3389/fnins.2021.683122

**Published:** 2021-06-08

**Authors:** K. A. Staats

**Affiliations:** ^1^Eli and Edythe Broad Center of Stem Cell Biology and Regenerative Medicine, Keck School of Medicine, University of Southern California, Los Angeles, CA, United States; ^2^Staats Life Sciences Consulting, LLC, Los Angeles, CA, United States

**Keywords:** stem cells, neurons, neurodegeneration, disease modeling, therapeutics

With the increasing number of people with neurodegenerative disease world-wide, novel directions and paradigms are sought to understand disease-specific neuronal death and to offer novel therapeutic strategies to patients. The ability to transform a differentiated cell into a pluripotent state and to differentiate again to a defined target cell type (Takahashi and Yamanaka, 2006) has led to the hope of the development of novel therapeutics. Although this holds promise in many fields, there is an extra challenge to replace any lost post-mitotic cells, such as neurons in neurodegeneration. With the development of replacement therapy for certain neuronal subtypes, e.g. dopaminergic neurons in models of Parkinson's Disease (PD) (Wakeman et al., 2017), there are remaining challenges with the long axons grown throughout development, e.g., in motor neurons in diseases such as Amyotrophic Lateral Sclerosis (ALS). Despite that stem cell-derived neurons may not always be suitable for replacement therapies, they are extremely informative in disease modeling to understand more of human disease as well as to discover and develop novel therapeutics. This Research Topic has included original and review papers spanning both topics, therapeutics and disease modeling, and discuss a large number of neuronal cell types.

In the context of ALS and Frontal Temporal Dementia (FTD), Guo et al. summarize the disease-specific phenotypes in patient-derived IPSC-derived neurons and the variability between reports (Guo et al.), identifying the limitations of these approaches, including the variable genetic backgrounds, off-target effects of genetic corrections or targeting, the lacking cellular maturity, and the heterogeneity of differentiation techniques. This latter point is further detailed by Ghaffari et al. whom provide an impressive deep-dive into the different means of differentiation of specific neuronal and glial subtypes in detail, and compare and contrast IPSCs and direct conversion. Despite these challenges, the use of patient-derived tissue is strongly recommended in disease-modeling and preclinical ALS studies for drug discovery and development (van den Berg et al., 2019). Besides disease modeling and the replacement of neurons, stem cell derived cells can also be developed as a therapeutic strategy to support neuronal survival, instead of adopting a neuronal fate. A summary of these strategies and their (pre)clinical support in ALS is provided by the article from Forostyak and Sykova, in which they outline the terminology and cell types that have been published. In particular, they highlight the protective effects on motor neurons that mesenchymal stromal cells offer by producing neurotrophic factors upon transplantation (Forostyak and Sykova).

With the epigenetic markers of cellular maturity lost when cells are converted to iPSCs (Mertens et al., 2015; Traxler et al., 2019), it can be challenging to detect late onset disease-specific pathology in iPSC-derived neurons. Seminary et al., recapitulated an impaired heat shock response in iPSC-derived motor neurons harboring ALS mutations, In addition, with this model they identified an accumulation of insoluble and aggregation-prone proteins, and that the presence of these was not sufficient to induce a heat shock response or stress-granule formation (Seminary et al.). The importance of how a gene may cause disease and whether that mechanism remains present in iPSC-derived neurons is also of relevance in the context of the regulation of the gene *SNCA*, encoding for the protein alpha-synuclein, in Parkinson's Disease (Piper et al.). Piper et al. describe in-depth the different ways *SNCA* may genetically cause disease, as well as how *SCNA* may be regulated. The authors stress the importance of the understanding of temporal and cell type-specific regulation of SNCA in disease, and in disease-models (Piper et al.).

In Huntington's disease (HD), this Research Topic's contributions span the increased understanding of pathophysiology, testing of novel therapeutics, and the transplantation of cells as a potential therapeutic.

Naphade et al. used patient-derived, isogenic, and control-corrected IPSCs to generate neural stem cells to assess the role of matrix metalloproteinases (MMPs) and their inhibitors in HD (Naphade et al.). They found that MMPs' endogenous inhibitors are decreased in HD cells and are elevated by TGFb treatment (Naphade et al.), illustrating a potential new direction for HD therapeutic strategies. Rindt et al. used a similar technique to assess the potential of the pre-mRNA repair of mutant Huntingtin, and also identified a beneficial response in HD-derived IPSC neural models (Rindt et al.). Subsequnetly, Masnata and Cicchetti describe the evidence for seeding of the Huntingtin protein in *in vitro* cultures of HD, including by IPSC disease modeling, and find sufficient evidence to suggest that this occurs, prompting the conclusion that *in vivo* assessment is now needed to further assess this (Masnata and Cicchetti). Al-Gharaibeh et al. transplanted IPSC-derived neural stem cells as a potential therapeutic into the striata of aged HD model mice (YAC128) and observed a striking protective effect on pathology and behavior in these animals (Al-Gharaibeh et al.), indicating the potential for neuronal replacement therapy in HD.

To understand the potential and limitations of the use of non-primary neurons, Drouin-Ouellet et al. summarize the use of inducible neurons (iNeurons), which are derived from direct differentiation from somatic cells (Drouin-Ouellet et al.). They delineate what constitutes an iNeuron, describe the benefits of each stage of differentiation per neurological disease, discuss whether generating subtype-specific iNeurons is critical to the disease-related features of these cells, and subsequently explain the biomedical potential and limitations of the use of these cells (Drouin-Ouellet et al.). In addition, Omais et al. summarize an alternative potential cells to model neurodegenerative disease; adult neurogenesis in the olfactory bulb (Omais et al.).

To accelerate the discovery and development of novel therapeutic strategies for patients, the Montreal Neurological Institute has adopted an “Open Science” model, as described by Han et al. Open Science refers to the transparency of the research and is illustrated by Open Lab Notebooks Durcan, and the sharing of resources, including patient-derived stem cell lines (Gan-Or et al., 2020). Such initiatives will hopefully provide novel ideas and collaborations, with improved disease modeling and therapeutic development for neurodegenerative diseases.

## Author Contributions

The author confirms being the sole contributor of this work and has approved it for publication.

## Conflict of Interest

KS is employed by company Staats Life Sciences Consulting, LLC.
